# Trends in COVID-19 cases and clinical management in Veterans Health Administration medical facilities: A national cohort study

**DOI:** 10.1371/journal.pone.0246217

**Published:** 2021-07-29

**Authors:** Maya Aboumrad, Brian Shiner, Natalie Riblet, Hugh Huizenga, Nabin Neupane, Yinong Young-Xu

**Affiliations:** 1 White River Junction Veterans Affairs Medical Center, White River Junction, Vermont, United States of America; 2 Department of Psychiatry, Geisel School of Medicine at Dartmouth, Hanover, New Hampshire, United States of America; University of Tripoli, LIBYA

## Abstract

**Objective:**

We explored longitudinal trends in sociodemographic characteristics, reported symptoms, laboratory findings, pharmacological and non-pharmacological treatment, comorbidities, and 30-day in-hospital mortality among hospitalized patients with coronavirus disease 2019 (COVID-19).

**Methods:**

This retrospective cohort study included patients diagnosed with COVID-19 in the United States Veterans Health Administration between 03/01/20 and 08/31/20 and followed until 09/30/20. We focused our analysis on patients that were subsequently hospitalized, and categorized them into groups based on the month of hospitalization. We summarized our findings through descriptive statistics. We used Cuzick’s Trend Test to examine any differences in the distribution of our study variables across the six months.

**Results:**

During our study period, we identified 43,267 patients with COVID-19. A total of 8,240 patients were hospitalized, and 13.1% (N = 1,081) died within 30 days of admission. Hospitalizations increased over time, but the proportion of patients that died consistently declined from 24.8% (N = 221/890) in March to 8.0% (N = 111/1,396) in August. Patients hospitalized in March compared to August were younger on average, mostly black, urban-dwelling, febrile and dyspneic. They also had a higher frequency of baseline comorbidities, including hypertension and diabetes, and were more likely to present with abnormal laboratory findings including low lymphocyte counts and elevated creatinine. Lastly, there was a decline from March to August in receipt of mechanical ventilation (31.4% to 13.1%) and hydroxychloroquine (55.3% to <1.0%), while treatment with dexamethasone (3.7% to 52.4%) and remdesivir (1.1% to 38.9%) increased.

**Conclusion:**

Among hospitalized patients with COVID-19, we observed a trend towards decreased disease severity and mortality over time.

## Introduction

Coronavirus disease 2019 (COVID-19) is an infectious respiratory illness caused by the severe acute respiratory syndrome coronavirus 2 (SARS-CoV-2) [1, 2]. As of February 28, 2021, the United States (U.S.) has the highest number of reported COVID-19 cases (28.4 million) and deaths (510,777) globally [2]. There is a growing body of literature utilizing electronic medical record data to provide “real-world” information on risk factors for SARS-CoV-2 infection and associated disease severity and mortality including certain sociodemographic characteristics, symptoms, laboratory findings, underlying comorbidities, and treatments [3–11]. However, the results of these studies are largely conflicting, especially among those conducted during the initial onset of the pandemic compared to more recent literature, as well as among those that were regional or local compared to national in scope [3–11].

Conflicting findings from “real-world” studies may be due to lack of consideration for the temporal and regional distribution of COVID-19 burden across the U.S., secular trends in clinical practice, or potential systemic differences in the characteristics of patients requiring hospitalization or treatment [6, 9, 12]. Moreover, the aggregation of this information at one time point and short-term assessment (e.g., one month) may not accurately account for the influence of rapidly deployed and changing mitigation efforts including government restrictions and policies [6, 13–16]. Therefore, longitudinal assessment of changes over multiple time points would provide greater clarity on the epidemiology and clinical management of COVID-19 and allow for indirect assessment of mitigation efforts.

To address this knowledge gap, we explored potential trends and patterns in COVID-19 case rates among all U.S. Veterans Health Administration (VHA) users, and clinical management for those that were subsequently hospitalized at VHA medical facilities during the initial six months of the pandemic. Specifically, our objectives were to describe any changes from March 1, 2020 to August 30, 2020 in 1) sociodemographic characteristics, 2) reported symptoms, 3) laboratory findings, 4) pharmacological and non-pharmacological treatment, 5) 30-day in-hospital mortality, and 6) comorbidities among both hospitalized and in-hospital deceased patients. As the nation’s largest integrated health care system, the VHA was required to respond to COVID-19 in all geographic regions [14, 15]. Thus, the VHA offers a unique opportunity to better understand the case burden and management of COVID-19 due to its large size, diverse operating environment, nationally overseen policies and practices, and relatively homogenous patient population [13–15]. Overall, the results of this study may be used to help inform ongoing relief efforts and improve future responses to novel diseases.

## Methods

This study received institutional review board approval from the Veteran’s Institutional Review Board of Northern New England at the White River Junction Veterans Affairs Medical Center. All study procedures were carried out in compliance with federal and institutional ethical guidelines. Sensitive patient-level data were anonymized, and only aggregated results are reported as set forth by VHA’s data use and protection requirements (VHA Directive 1200.12). The requirement to obtain informed consent from study participants was waived as the Institutional Review Board deemed this study to involve no more than a minimal risk to the privacy of individuals. Our study was reported in accordance with the Strengthening the Reporting of Observational Studies in Epidemiology (STROBE) guidelines (S1 Checklist).

### Data source & study population

The VHA is comprised of over 170 medical centers and 1,250 community-based outpatient clinics [17]. It provides comprehensive medical care to more than nine million veterans, including primary and specialty care [17]. Additionally, VHA users have access to extensive inpatient care and treatment services including medical, surgical, mental health, dialysis, acute and long-term care [17]. The VHA has an electronic medical record system with a centralized Corporate Data Warehouse, which contains longitudinal information on receipt of all services provided by VHA facilities including outpatient and inpatient visits, pharmacological and non-pharmacological treatments, and laboratory results, as well as patients’ sociodemographic and clinical characteristics. We obtained patients’ vital status (i.e., alive or date of death if applicable) from the VHA Vital Status File.

Our final cohort consisted of all VHA users (age ≥ 18 years) diagnosed with COVID-19 between March 1, 2020 and August 31, 2020 in accordance with Centers for Disease Control and Prevention standards, human confirmed case review, and/or VHA guidelines (10N memos) [13, 18].

### Outcomes

Hospitalization: We limited our reporting to a subset of patients that were subsequently hospitalized for COVID-19 within the VHA health care system due to lack of complete clinical and outcome data for patients that received care at a non-VHA facility. Patients were indexed at the time of hospitalization for COVID-19 and categorized into six groups based on the month of hospitalization. We calculated hospital length of stay (LOS) as the number of days from admission to discharge or in-hospital death.

Mortality: Our primary endpoint of interest was 30-day in-hospital mortality. We examined mortality within 30 days of hospital admission to serve as a proxy for case mortality rate [4]. We extended patient follow-up to September 30, 2020 to allow for sufficient assessment time of our study measures. Patients remained in the cohort until date of discharge, in-hospital death, or end of 30-day follow-up, whichever occurred first.

### Study variables

#### Patient characteristics

We examined sociodemographic characteristics at the time of hospitalization including age, sex, race, urbanicity of residence, and VHA priority rating (1–8). VHA priority group served as a proxy for socioeconomic status (SES) as it is partially based on income and the capacity for gainful employment. It is also connected to service-related disability, reflecting both health and VHA coverage [19]. Priority group ratings range from 1–8, with group 1 representing the highest priority (i.e., lowest SES and complete healthcare coverage). Additionally, we reported the race of in-hospital deceased patients due to prior work documenting elevated mortality rates among racial minorities with COVID-19 [5, 7].

#### Symptoms

We examined the presence of COVID-related symptoms within 30 days preceding hospitalization through a combination of International Classification of Diseases, Tenth Revision (ICD-10) codes and natural language processing of clinician notes in patients’ electronic medical records. These included abdominal pain, chills, common cold, cough, diarrhea, dyspnea, fatigue, fever, headache, myalgia, nausea, and sore throat [5, 10, 20].

#### Laboratory values

We examined laboratory values within seven days following hospitalization including white blood cell count (WBC), absolute lymphocyte count (LC), platelet count (PC), creatinine, blood urea nitrogen (BUN), total bilirubin, aspartate aminotransferase (AST), alanine aminotransferase (ALT), ferritin, lactate, troponin I, brain-type natriuretic peptide (BNP), procalcitonin, c-reactive protein (CRP) [5, 10, 20]. If more than one value was available, we selected the value closest to the time of hospitalization.

#### Treatments

We examined receipt of any relevant/concomitant pharmacological and non-pharmacological treatment(s) during the course of patients’ hospitalization. Pharmacological treatments included angiotensin-converting enzyme (ACE) inhibitors, antibiotics (e.g., azithromycin), anticoagulants, hydroxychloroquine, remdesivir, azithromycin combined with hydroxychloroquine, beta-blockers, bronchodilators, corticosteroids (e.g., dexamethasone), immune-based therapy (e.g., tocilizumab), non-steroidal anti-inflammatory drugs (NSAIDs), statins, and/or vasopressors [21, 22]. Non-pharmacological treatments included mechanical ventilation, dialysis, and supplemental oxygen.

#### Comorbidities

We examined the presence of clinical comorbidities within 12 months preceding hospitalization using ICD-10 diagnosis codes recorded in patients’ electronic medical records and categorized them according to the Charlson Comorbidity Index (CCI). The CCI score is a validated, weighted measure that predicts one-year mortality, with higher scores indicating greater illness burden [23]. Additionally, we reported the top 10 comorbidities for both hospitalized and in-hospital deceased patients.

### Statistical analysis

For all VHA users with COVID-19, we reported the frequency of cases by month of diagnosis to examine trends in overall case rates. For patients that were subsequently hospitalized for COVID-19 within the VHA, we reported our findings by month of hospitalization to examine trends in clinical management during the course of the pandemic.

We summarized our results through descriptive statistics. We reported proportion for categorical variables, and mean (M) with standard deviation (SD) and median with interquartile range (IQR) for continuous variables. We report the proportion of missing data where applicable. We used Cuzick’s Trend Test, a nonparametric rank-sum test for trend, to examine any differences in the distribution of our study variables across the six months. All analyses were performed using Stata/MP version 15.1 software (StataCorp, 2015).

## Results

### Frequency of COVID-19 cases among all VHA users

We identified a total of 43,267 VHA users with COVID-19 during our study period. Of these cases, 5.4% (N = 2,344) were diagnosed in March, 16.1% (N = 6,947) in April, 10.6% (N = 4,594) in May, 15.9% (N = 6,867) in June, 33.2% (N = 14,369) in July, and 18.8% (N = 8,146) in August. There was a significant trend towards increasing COVID-19 cases reported from March to August (P_trend_<0.001).

### Patient characteristics among VHA users hospitalized with COVID-19

Our cohort comprised 8,240 patients hospitalized with COVID-19 (Table 1). The average and median LOS was 10.2 days (SD = 10.9) and 6.0 days (IQR = 10.0), respectively. The proportion of hospitalized patients increased from 10.8% (N = 890) in March to 16.9% (N = 1,396) in August (P_trend_<0.001). Months with the highest proportion of hospitalized patients were April (N = 1,559, 18.9%) and July (N = 2,279, 27.7%). Among hospitalized patients, 13.1% (N = 1,081) died within 30-days of admission. The proportion of patients that experienced 30-day in-hospital mortality consistently declined from 24.8% (N = 221/890) in March to 8.0% (N = 111/1,396) in August (P_trend_<0.001). Black patients represented 37.7% (N = 407/1,081) of in-hospital deaths overall, the proportion of which consistently declined from 60.2% (N = 133/221) in March to 19.8% (N = 22/111) in August (P_trend_<0.001).

**Table 1:** Patient characteristics* among 8,240 hospitalized Veterans Health Administration users with coronavirus disease 2019 (COVID-19), March 1, 2020 –August 31, 2020.

	March N (%)	April N (%)	May N (%)	June N (%)	July N (%)	August N (%)	Total N (%)	P-trend
**Total Hospitalized**	890 (10.8)	1,559 (18.9)	889 (10.8)	1,227 (14.9)	2,279 (27.7)	1,396 (16.9)	8,240 (100.0)	<0.001
Age in years								0.001
≤49	78 (8.8)	101 (6.5)	68 (7.7)	159 (13.0)	255 (11.2)	126 (9.0)	787 (9.6)	
50–59	148 (16.6)	171 (11.0)	94 (10.6)	181 (14.8)	326 (14.3)	179 (12.8)	1,099 (13.3)	
60–69	235 (26.4)	385 (24.7)	250 (28.1)	304 (24.8)	591 (25.9)	344 (24.6)	2,109 (25.6)	
70–79	288 (32.4)	515 (33.0)	273 (30.7)	390 (31.8)	743 (32.6)	475 (34.0)	2,684 (32.6)	
≥80	141 (15.8)	387 (24.8)	204 (23.0)	193 (15.7)	364 (16.0)	272 (19.5)	1,561 (18.9)	
Mean (SD)	67.4 (13.0)	70.7 (13.1)	69.9 (13.5)	66.4 (14.7)	67.0 (13.8)	68.4 (13.5)	68.2 (13.7)	0.001
Median (IQR)	69.0 (16.0)	71.0 (16.0)	70.0 (15.0)	69.0 (17.0)	69.0 (16.0)	71.0 (15.0)	70.0 (16.0)	0.001
Male sex	853 (95.8)	1,490 (95.6)	846 (95.2)	1,148 (93.6)	2,147 (94.2)	1,315 (94.2)	7,799 (94.7)	0.01
Race								<0.001
White	313 (35.2)	751 (48.2)	465 (52.3)	669 (54.5)	1,242 (54.5)	828 (59.3)	4,268 (51.8)	
Black	508 (57.1)	703 (45.1)	354 (39.8)	438 (35.7)	813 (35.7)	431 (30.9)	3,247 (39.4)	
Other	69 (7.8)	105 (6.7)	70 (7.9)	120 (9.8)	224 (9.8)	137 (9.8)	725 (8.8)	
Urban residence	837 (94.0)	1,426 (91.5)	750 (84.4)	1,018 (83.0)	1,830 (80.3)	954 (68.3)	6,815 (82.7)	<0.001
Priority Rating (1–8)								0.56
1–4	266 (29.9)	450 (28.9)	260 (29.3)	339 (27.6)	649 (28.5)	408 (29.2)	2,372 (28.8)	
5–6	320 (36.0)	577 (37.0)	345 (38.8)	509 (41.5)	912 (40.0)	526 (37.7)	3,189 (38.7)	
7–8	304 (34.2)	532 (34.1)	284 (32.0)	379 (30.9)	718 (31.5)	462 (33.1)	2,679 (32.5)	
Length of stay in days								
Mean (SD)	10.1 (10.9)	10.8 (11.9)	11.8 (12.4)	10.6 (11.7)	9.6 (9.9)	9.3 (9.1)	10.2 (10.9)	<0.001
Median (IQR)	6.0 (9.0)	6.0 (10.0)	7.0 (12.0)	6.0 (10.0)	6.0 (9.0)	6.0 (9.0)	6.0 (10.0)	<0.001
30-day in-hospital mortality	221 (24.8)	325 (20.8)	103 (11.6)	115 (9.4)	206 (9.0)	111 (8.0)	1,081 (13.1)	<0.001
*White*	76 (34.4)	158 (48.6)	57 (55.3)	67 (58.3)	128 (62.1)	77 (69.4)	563 (52.1)	<0.001
*Black*	133 (60.2)	135 (41.5)	30 (29.1)	36 (31.3)	51 (24.8)	22 (19.8)	407 (37.7)	<0.001
*Other*	12 (5.4)	32 (9.8)	16 (15.5)	12 (10.4)	27 (13.1)	12 (10.8)	111 (10.3)	<0.001

*Socio-demographic characteristics were examined at the time of hospitalization for COVID-19.

**Note.** N = Number; SD = Standard Deviation; IQR = Interquartile Range; suppressed = suppressed values for patient privacy due to too few counts and/or back-calculation possible.

Overall, hospitalized patients were older (M = 68.2, SD = 13,7; Median = 70.0, IQR = 16.0), predominantly male (N = 7,799, 94.7%), and white (N = 4,268, 51.8%). The majority resided in an urban area (N = 6,815, 82.7%), and close to 40% (N = 3,189) had a priority rating of 5–6. Both the average and median age of hospitalized patients increased from March (M = 67.4, SD = 13.0; Median = 69.0, IQR = 16.0) to August (M = 68.4, SD = 13.5; Median = 71.0, IQR = 15.0), and patients aged 60 years and older consistently represented at least 70% of hospitalizations (P_trend_ = 0.001). Months with a greater proportion of younger (age <60 years) patients included March, June, and July. Black patients represented 57.1% (N = 508/890) of hospitalizations in March, but consistently declined to 30.9% (N = 431/1,396) in August (P_trend_<0.001). The proportion of hospitalized patients with urban residence consistently declined from 94.0% (N = 837/890) in March to 68.3% (N = 954/1,396) in August (P_trend_<0.001).

### COVID-19 symptoms

The most frequently reported symptoms among hospitalized patients were fever (N = 3,967, 48.1%), dyspnea (N = 2,398, 29.1%), and cough (N = 1,705, 20.7%) (Table 2). The proportion of patients reporting these symptoms significantly declined from March to August (P_trend_<0.001).

**Table 2:** Patient symptoms* among 8,240 hospitalized Veterans Health Administration users with coronavirus disease 2019 (COVID-19), March 1, 2020 –August 31, 2020.

	March N (%)	April N (%)	May N (%)	June N (%)	July N (%)	August N (%)	Total N (%)	P-trend
**Total Hospitalized**	890 (10.8)	1,559 (18.9)	889 (10.8)	1,227 (14.9)	2,279 (27.7)	1,396 (16.9)	8,240 (100.0)	<0.001
Fever	606 (68.1)	807 (51.8)	394 (44.3)	557 (45.4)	1,030 (45.2)	573 (41.1)	3,967 (48.1)	<0.001
Dyspnea	361 (40.6)	482 (30.9)	216 (24.3)	322 (26.2)	650 (28.5)	367 (26.3)	2,398 (29.1)	<0.001
Cough	344 (38.7)	389 (25.0)	128 (14.4)	218 (17.8)	408 (17.9)	218 (15.6)	1,705 (20.7)	<0.001
Fatigue	139 (15.6)	217 (13.9)	100 (11.3)	172 (14.0)	279 (12.2)	195 (14.0)	1,102 (13.4)	0.19
Common cold	202 (22.7)	157 (10.1)	54 (6.1)	94 (7.7)	155 (6.8)	90 (6.5)	752 (9.1)	<0.001
Diarrhea	91 (10.2)	135 (8.7)	72 (8.1)	85 (6.9)	175 (7.7)	96 (6.9)	654 (7.9)	0.003
Nausea	64 (7.2)	81 (5.2)	47 (5.3)	73 (6.0)	128 (5.6)	90 (6.5)	483 (5.9)	0.97
Abdominal pain	49 (5.5)	61 (3.9)	46 (5.2)	57 (4.7)	108 (4.7)	68 (4.9)	389 (4.7)	0.88
Headache	23 (2.6)	32 (2.1)	23 (2.6)	24 (2.0)	55 (2.4)	32 (2.3)	189 (2.3)	0.99
Chills	39 (4.4)	24 (1.5)	18 (2.0)	27 (2.2)	42 (1.8)	19 (1.4)	169 (2.1)	0.001
Myalgia	24 (2.7)	13 (1.0)	suppressed	suppressed	25 (1.1)	suppressed	91 (1.1)	0.001
Sore throat	15 (1.7)	suppressed	suppressed	suppressed	23 (1.0)	16 (1.2)	80 (1.0)	0.66

*COVID-19 symptoms were examined within 30 days preceding hospitalization.

**Note.** N = Number; suppressed = suppressed values for patient privacy due to too few counts and/or back-calculation possible.

### Clinical comorbidities among hospitalized and in-hospital deceased patients

Hospitalized patients had an average CCI score of 1.8 (SD = 2.1) and median of 1.0 (IQR = 3.0) (Table 3). Those that experienced 30-day in-hospital mortality had an average CCI score of 2.3 (SD = 2.3) and median of 2.0 (IQR = 4.0). CCI score significantly declined over time for both hospitalized (P_trend_<0.001) and in-hospital deceased (P_trend_ = 0.02) patients.

**Table 3:** Top 10 clinical comorbidities* among hospitalized and in-hospital deceased Veterans Health Administration users with coronavirus disease 2019 (COVID-19), March 1, 2020 –August 31, 2020.

	March N (%)	April N (%)	May N (%)	June N (%)	July N (%)	August N (%)	Total N (%)	P-trend
**Total Hospitalized**	890 (10.8)	1,559 (18.9)	889 (10.8)	1,227 (14.9)	2,279 (27.7)	1,396 (16.9)	8,240 (100.0)	<0.001
CCI Score								
Mean (SD)	2.0 (2.3)	2.1 (2.2)	2.0 (2.2)	1.7 (2.0)	1.6 (2.0)	1.6 (1.9)	1.8 (2.1)	<0.001
Median (IQR)	1.0 (3.0)	1.0 (3.0)	1.0 (3.0)	1.0 (3.0)	1.0 (3.0)	1.0 (3.0)	1.0 (3.0)	<0.001
Hypertension	574 (64.5)	973 (62.4)	496 (55.8)	658 (53.6)	1,201 (52.7)	742 (53.2)	4,644 (56.4)	<0.001
Diabetes	370 (41.6)	628 (40.3)	358 (40.3)	434 (35.4)	854 (37.5)	526 (37.7)	3,170 (38.5)	0.01
CAD	191 (21.5)	334 (21.4)	169 (19.0)	228 (18.6)	395 (17.3)	249 (17.8)	1,566 (19.0)	<0.001
Renal disease	185 (20.8)	360 (23.1)	176 (19.8)	207 (16.9)	353 (15.5)	228 (16.3)	1,509 (18.3)	<0.001
COPD	162 (18.2)	350 (22.5)	164 (18.5)	187 (15.2)	346 (15.2)	244 (17.5)	1,453 (17.6)	<0.001
CHF	146 (16.4)	292 (18.7)	143 (16.1)	153 (12.5)	290 (12.7)	172 (12.3)	1,196 (14.5)	<0.001
Obesity	160 (18.0)	228 (14.6)	123 (13.8)	157 (12.8)	302 (13.3)	187 (13.4)	1,157 (14.0)	0.003
PVD	99 (11.1)	204 (13.1)	113 (12.7)	117 (9.5)	195 (8.6)	132 (9.5)	860 (10.4)	<0.001
Dementia	93 (10.5)	230 (14.8)	119 (13.4)	120 (9.8)	165 (7.2)	129 (9.2)	856 (10.4)	<0.001
Cancer	92 (10.3)	155 (9.9)	72 (8.1)	117 (9.5)	179 (7.9)	132 (9.5)	747 (9.1)	0.11
**Total In-hospital Deceased**	221 (20.4)	325 (30.1)	103 (9.5)	115 (10.6)	206 (19.1)	111 (10.3)	1,081 (100.0)	<0.001
CCI Score								
Mean (SD)	2.5 (2.4)	2.5 (2.3)	2.4 (2.7)	1.9 (2.1)	2.0 (2.1)	2.1 (2.0)	2.3 (2.3)	0.02
Median (IQR)	2.0 (4.0)	2.0 (4.0)	2.0 (3.5)	1.0 (3.0)	2.0 (3.0)	2.0 (3.0)	2.0 (4.0)	0.02
Hypertension	163 (73.8)	206 (63.4)	51 (49.5)	55 (47.8)	126 (61.2)	61 (55.0)	662 (61.2)	<0.001
Diabetes	108 (48.9)	136 (41.9)	41 (39.8)	48 (41.7)	101 (49.0)	48 (43.2)	482 (44.6)	0.91
Renal disease	54 (24.4)	102 (31.4)	21 (20.4)	28 (24.4)	51 (24.8)	27 (24.3)	283 (26.2)	0.33
CAD	59 (26.7)	85 (26.2)	21 (20.4)	23 (20.0)	54 (26.2)	23 (20.7)	265 (24.5)	0.20
COPD	46 (20.8)	85 (26.2)	17 (16.5)	11 (9.6)	41 (19.9)	19 (17.1)	219 (20.3)	0.03
CHF	48 (21.7)	81 (24.9)	20 (19.4)	11 (9.6)	33 (16.0)	17 (15.3)	210 (19.4)	0.002
Dementia	23 (10.4)	61 (18.8)	23 (22.3)	21 (18.3)	23 (11.2)	18 (16.2)	169 (15.6)	0.82
PVD	33 (14.9)	56 (17.2)	13 (12.6)	suppressed	24 (11.7)	suppressed	146 (13.5)	0.03
Obesity	37 (16.7)	53 (16.3)	suppressed	suppressed	32 (15.5)	suppressed	144 (13.3)	0.01
CVD	29 (13.1)	44 (13.5)	16 (15.5)	suppressed	25 (12.1)	suppressed	131 (12.1)	0.25

**Note.** N = Number; SD = Standard Deviation; IQR = Interquartile Range; CCI = Charlson Comorbidity Index; CAD = Coronary Artery Disease; COPD = Chronic Obstructive Pulmonary Disease; CHF = Congestive Heart Failure; PVD = Peripheral Vascular Disease; CVD = Cerebrovascular Disease; suppressed = suppressed values for patient privacy due to too few counts and/or back-calculation possible.

*Clinical comorbidities were examined within 12 months preceding hospitalization for COVID-19.

The top 10 comorbidities among hospitalized patients were hypertension (N = 4,644, 56.4%), diabetes (N = 3,170, 38.5%), coronary artery disease (N = 1,566, 19.0%), renal disease (N = 1,509, 18.3%), chronic obstructive pulmonary disease (N = 1,453, 17.6%), congestive heart failure (N = 1,196, 14.5%), obesity (N = 1,157, 14.0%), peripheral vascular disease (N = 860, 10.4%), dementia (N = 856, 10.4%), and cancer (N = 747, 9.1%). There was a significant decline from March to August in the proportion of hospitalized patients with hypertension (P_trend_<0.001), diabetes (P_trend_<0.01), coronary artery disease (P_trend_<0.001), renal disease (P_trend_<0.001), chronic obstructive pulmonary disease (P_trend_<0.001), congestive heart failure (P_trend_<0.001), obesity (P_trend_ = 0.003), peripheral vascular disease (P_trend_<0.001), and dementia (P_trend_<0.001). There was no significant trend change from March to August in the proportion of hospitalized patients with cancer (P_trend_ = 0.11).

The top 10 comorbidities among in-hospital deceased patients were similar to those reported among hospitalized patients. These included hypertension (N = 662, 61.2%), diabetes (N = 482, 44.6%), renal disease (N = 283, 26.2%), coronary artery disease (N = 265, 24.5%), chronic obstructive pulmonary disease (N = 219, 20.3%), congestive heart failure (N = 210, 19.4%), dementia (N = 169, 15.6%), peripheral vascular disease (N = 146, 13.5%), obesity (N = 144, 13.3%), and cerebrovascular disease (N = 131, 12.1%). There was a significant decline from March to August in the proportion of in-hospital deceased patients with hypertension (P_trend_<0.001), chronic obstructive pulmonary disease (P_trend_ = 0.03), congestive heart failure (P_trend_ = 0.002), peripheral vascular disease (P_trend_ = 0.03), and obesity (P_trend_ = 0.003). There were no significant trend changes from March to August in the proportion of in-hospital deceased patients with diabetes (P_trend_ = 0.91), renal disease (P_trend_ = 0.33), coronary artery disease (P_trend_ = 0.20), dementia (P_trend_ = 0.82), and cerebrovascular disease (P_trend_ = 0.25).

### Laboratory values among VHA users hospitalized with COVID-19

WBC, PC, and LC were low for 15.5% (N = 1,280), 18.4% (N = 1,517), and 41.9% (N = 3,453) of hospitalized patients, respectively (Table 4). Over one-third of patients had elevated creatinine (N = 3,153, 38.3%), BUN (N = 2,804, 34.0%), AST (N = 2,746, 33.3%), ferritin (N = 3,614, 43.9%), and lactate (N = 3,576, 43.4%). More than one-half (N = 4,237, 51.4%) of patients had elevated CRP. For almost all laboratory values, patients hospitalized in March either had a similar or greater proportion of abnormal results compared to those hospitalized in later months. However, the proportion of patients with elevated CRP increased from 49.6% (N = 441/890) in March to 54.3% (N = 758/1,396) in August (P_trend_<0.001).

**Table 4:** Laboratory values* among 8,240 hospitalized Veterans Health Administration users with coronavirus disease 2019 (COVID-19), March 1, 2020 –August 31, 2020.

	March N (%)	April N (%)	May N (%)	June N (%)	July N (%)	August N (%)	Total N (%)	P-trend
**Total Hospitalized**	890 (10.8)	1,559 (18.9)	889 (10.8)	1,227 (14.9)	2,279 (27.7)	1,396 (16.9)	8,240 (100.0)	<0.001
WBC <4,000/μL	151 (17.0)	248 (15.9)	119 (13.4)	177 (14.4)	363 (15.9)	222 (15.9)	1,280 (15.5)	0.24
Unknown/missing	90 (10.5)	131 (8.4)	67 (7.5)	94 (7.7)	174 (7.6)	122 (8.7)	681 (8.3)	
Absolute LC <1,000/μL	438 (49.2)	705 (45.2)	362 (40.7)	451 (36.8)	916 (40.2)	581 (41.6)	3,453 (41.9)	0.13
Unknown/missing	42 (4.7)	81 (5.2)	50 (5.6)	95 (7.7)	163 (7.2)	104 (7.5)	535 (6.5)	
PC <150,000/μL	194 (21.8)	296 (19.0)	132 (14.9)	216 (17.6)	429 (18.8)	250 (17.9)	1,517 (18.4)	0.28
Unknown/missing	34 (3.8)	72 (4.6)	45 (5.1)	74 (6.0)	143 (6.3)	91 (6.5)	459 (5.6)	
Creatinine >1.5 mg/dL	380 (42.7)	633 (40.6)	333 (37.5)	440 (35.9)	874 (38.4)	493 (35.3)	3,153 (38.3)	<0.001
Unknown/missing	35 (3.9)	68 (4.4)	45 (5.1)	71 (5.8)	140 (6.1)	90 (6.5)	449 (5.5)	
BUN >20 mg/dL	335 (37.6)	624 (40.0)	343 (38.6)	372 (30.3)	691 (30.3)	439 (31.5)	2,804 (34.0)	<0.001
Unknown/missing	35 (3.9)	68 (4.4)	45 (5.1)	71 (5.8)	140 (6.1)	90 (6.5)	449 (5.5)	
Total bilirubin ≥1.2 mg/dL	47 (5.3)	104 (6.7)	41 (4.6)	67 (5.5)	164 (7.2)	81 (5.8)	504 (6.1)	0.05
Unknown/missing	41 (4.6)	82 (5.3)	61 (6.9)	90 (7.3)	161 (7.1)	111 (8.0)	546 (6.6)	
AST >40 U/L	383 (43.0)	606 (38.9)	269 (30.3)	342 (27.9)	742 (32.6)	404 (28.9)	2,746 (33.3)	<0.001
Unknown/missing	49 (5.5)	97 (6.2)	68 (7.7)	104 (8.5)	181 (7.9)	131 (9.4)	630 (7.7)	
ALT >40 U/L	172 (19.3)	226 (14.5)	98 (11.0)	175 (14.3)	347 (15.2)	200 (14.3)	1,218 (14.8)	<0.001
Unknown/missing	47 (5.3)	95 (6.1)	63 (7.1)	103 (8.4)	180 (7.9)	134 (9.6)	622 (7.6)	
Ferritin >300 ng/mL	448 (50.3)	754 (48.4)	348 (39.2)	491 (40.0)	1,009 (44.3)	564 (40.4)	3,614 (43.9)	<0.001
Unknown/missing	265 (29.8)	375 (24.1)	230 (25.9)	343 (28.0)	600 (26.3)	425 (30.4)	2,238 (27.2)	
Lactate >2.2 mmol/L	465 (52.3)	747 (47.9)	342 (38.5)	488 (39.8)	987 (43.3)	547 (39.2)	3,576 (43.4)	<0.001
Unknown/missing	282 (31.7)	455 (29.2)	300 (33.8)	416 (33.9)	748 (32.8)	540 (38.7)	2,741 (33.3)	
Troponin I ≥0.06 ng/mL	148 (16.6)	233 (15.0)	105 (11.8)	126 (10.3)	297 (13.0)	179 (12.8)	1,088 (13.2)	0.31
Unknown/missing	268 (30.1)	482 (30.9)	302 (34.0)	349 (28.4)	641 (28.1)	457 (32.7)	2,499 (30.3)	
BNP >100 pg/mL	216 (24.3)	403 (25.9)	222 (25.0)	257 (21.0)	471 (20.7)	300 (21.5)	1,869 (22.7)	0.002
Unknown/missing	378 (42.5)	734 (47.1)	444 (49.9)	572 (46.6)	1,026 (45.0)	714 (51.2)	3,868 (46.9)	
Procalcitonin >0.25 ng/mL	212 (23.8)	437 (28.0)	188 (21.2)	184 (15.0)	373 (16.4)	218 (15.6)	1,612 (19.6)	<0.001
Unknown/missing	392 (44.0)	670 (43.0)	439 (49.4)	596 (48.6)	1,050 (46.1)	713 (51.1)	3,860 (46.8)	
CRP >8.2 ng/mL	441 (49.6)	785 (50.4)	441 (49.6)	579 (47.2)	1,233 (54.1)	758 (54.3)	4,237 (51.4)	<0.001
Unknown/missing	423 (47.5)	696 (44.6)	362 (40.7)	545 (44.4)	851 (37.3)	508 (36.4)	3,385 (41.1)	

**Note.** N = Number; WBC = White Blood Cell Count; LC = Lymphocyte Count; PC = Platelet Count; BUN = Blood Urea Nitrogen; AST = Aspartate Aminotransferase; ALT = Alanine Aminotransferase; BNP = Brain-type Natriuretic Peptide; CRP = C-reactive Protein; suppressed = suppressed values for patient privacy due to too few counts and/or back-calculation possible.

*Laboratory values were examined within seven days following hospitalization for COVID-19.

### Pharmacological and non-pharmacological COVID-19 treatment

Approximately one-third (N = 2,433, 29.5%) of hospitalized patients were treated with dexamethasone, the use of which significantly increased from 3.7% (N = 33/890) in March to 52.4% (N = 732/1,396) by August (P_trend_<0.001) (Table 5). One-quarter (N = 2,042, 24.8%) of hospitalized patients were treated with remdesivir. Treatment with remdesivir significantly increased from 1.1% (N = 10/890) in March to 38.9% (N = 543/1,396) in August (P_trend_<0.001). Less than one-fifth (N = 1,074, 13.0%) of hospitalized patients were treated with hydroxychloroquine. Treatment with hydroxychloroquine significantly declined from 55.3% (N = 492/890) in March to less than 1% in August (P_trend_<0.001). Very few hospitalized patients were treated with vasopressors (N = 1,006, 12.2%), the use of which significantly declined from 21.2% (N = 189/890) in March to 8.5% (N = 119/1,396) in August (P_trend_<0.001).

**Table 5:** Pharmacological and non-pharmacological treatment* among 8,240 hospitalized Veterans Health Administration users with coronavirus disease 2019 (COVID-19), March 1, 2020 –August 31, 2020.

	March N (%)	April N (%)	May N (%)	June N (%)	July N (%)	August N (%)	Total N (%)	P-trend
**Total Hospitalized**	890 (10.8)	1,559 (18.9)	889 (10.8)	1,227 (14.9)	2,279 (27.7)	1,396 (16.9)	8,240 (100.0)	<0.001
**Pharmacological Treatments**								
Anticoagulants	799 (89.8)	1,372 (88.0)	792 (89.1)	1,078 (87.9)	2,098 (92.1)	1,303 (93.3)	7,442 (90.3)	<0.001
Statins	527 (59.2)	894 (57.3)	515 (57.9)	693 (56.5)	1,342 (58.9)	796 (57.0)	4,767 (57.9)	0.78
Antibiotics	678 (76.2)	931 (59.7)	448 (50.4)	588 (47.9)	1,131 (49.6)	692 (49.6)	4,468 (54.2)	<0.001
Azithromycin^**^	510 (57.3)	514 (33.0)	215 (24.2)	280 (22.8)	632 (27.7)	349 (25.0)	2,500 (30.3)	<0.001
NSAIDs	455 (51.1)	771 (49.5)	470 (52.9)	661 (53.9)	1,239 (54.4)	797 (57.1)	4,393 (53.3)	<0.001
Beta-blockers	439 (49.3)	738 (47.3)	422 (47.5)	530 (43.2)	1,044 (45.8)	700 (50.1)	3,873 (47.0)	0.83
Corticosteroids	245 (27.5)	413 (26.5)	214 (24.1)	580 (47.3)	1,440 (63.2)	882 (63.2)	3,774 (45.8)	<0.001
Dexamethasone^**^	33 (3.7)	43 (2.8)	29 (3.3)	391 (31.9)	1,205 (52.9)	732 (52.4)	2,433 (29.5)	<0.001
Bronchodilators	397 (44.6)	687 (44.1)	348 (39.2)	552 (45.0)	1,043 (45.8)	632 (45.3)	3,659 (44.4)	0.15
Remdesivir	10 (1.1)	34 (2.2)	191 (21.5)	390 (31.8)	874 (38.4)	543 (38.9)	2,042 (24.8)	<0.001
ACE-inhibitors	183 (20.6)	315 (20.2)	195 (21.9)	296 (24.1)	596 (26.2)	343 (24.6)	1,928 (23.4)	<0.001
Hydroxychloroquine	492 (55.3)	514 (33.0)	27 (3.0)	suppressed	19 (1.0)	suppressed	1,074 (13.0)	<0.001
Vasopressors	189 (21.2)	256 (16.4)	113 (12.7)	123 (10.0)	206 (9.0)	119 (8.5)	1,006 (12.2)	<0.001
Azithromycin + hydroxychloroquine	370 (41.6)	294 (18.9)	suppressed	suppressed	14 (1.0)	suppressed	698 (8.5)	<0.001
Immune-based therapy	55 (6.2)	145 (9.3)	45 (5.1)	45 (3.7)	93 (4.1)	16 (1.2)	399 (4.8)	<0.001
Tocilizumab^**^	52 (5.8)	133 (8.5)	45 (5.1)	45 (3.7)	93 (4.1)	16 (1.2)	384 (4.7)	<0.001
**Non-pharmacological Treatments**								
Mechanical ventilation	279 (31.4)	330 (21.2)	150 (16.9)	165 (13.5)	354 (15.5)	183 (13.1)	1,461 (17.7)	<0.001
Dialysis	129 (14.5)	165 (10.6)	66 (7.4)	69 (5.6)	130 (5.7)	59 (4.2)	618 (7.5)	<0.001
Supplemental oxygen	67 (7.5)	137 (8.8)	57 (6.4)	82 (6.7)	178 (7.8)	90 (6.5)	611 (7.4)	0.16

**Note.** N = Number; NSAIDs = Non-steroidal Anti-inflammatory Drugs; ACE = Angiotensin-converting Enzyme; suppressed = suppressed values for patient privacy due to too few counts and/or back-calculation possible.

*Receipt of any pharmacological and/or non-pharmacological treatment(s) was examined during the course of hospitalization for COVID-19.

**Included in count of parent treatment category.

Close to one-fifth (N = 1,461, 17.7%) of hospitalized patients received mechanical ventilation, the use of which significantly declined from 31.4% (N = 279/890) in March to 13.1% (N = 183/1,396) in August (P_trend_<0.001). A small proportion of hospitalized patients received dialysis (N = 618, 7.5%) and supplemental oxygen (N = 611, 7.4%). Treatment with dialysis declined from 14.5% (N = 129/890) in March to 4.2% (N = 59/1,396) in August (P_trend_<0.001), while use of supplemental oxygen remained constant over time (P_trend_ = 0.16).

## Discussion

To the best of our knowledge, this is the first study that examined longitudinal trends in COVID-19 case rates as well as clinical management within a national health care system. Within the VHA, COVID-19 cases and hospitalization rates fluctuated between March and August, but increased overall. We identified peaks for both cases and hospitalizations in April, July, and August. Patients hospitalized in March compared to later months were younger, mostly black and urban-dwelling, and more likely to present with fever, dyspnea, cough, and abnormal laboratory findings. They also had poorer overall health as indicated by higher CCI scores and frequency of baseline comorbidities. Lastly, we observed a decline in use of intensive (e.g., mechanical ventilation) and experimental (e.g., hydroxychloroquine) treatments for COVID-19, as well as a consistent decline in 30-day in-hospital mortality over time.

Many prior studies have worked to identify risk factors for SARS-CoV-2 infection and disease severity and mortality [3–11]. Literature published early on in the pandemic, as well as more recent studies, have consistently reported an elevated risk of both testing positive and severe or fatal disease among older populations (age ≥65 years) after adjusting for sociodemographic characteristics and comorbidities [3, 6, 9]. In our study, we found that the majority of patients hospitalized with COVID-19 were older. However, we also identified a higher proportion of younger patients hospitalized in months with corresponding elevations in case rates. This finding may support the notion that young and presumably healthy individuals can contribute to the inadvertent spread of SARS-CoV-2 [6, 11]. Although we observed a statistically significant trend towards increasing age over time, our findings should be interpreted with caution as the magnitude of the change may not be clinically meaningful. Containing viral transmission may require more ubiquitous government restrictions and policies including testing, contact tracing, social distancing and work-from-home guidelines, as well as targeted interventions for particularly high-risk groups [6, 16]. Moreover, we observed a trend towards declining 30-day in-hospital mortality and treatment-related indicators of severe illness (e.g., receipt of mechanical ventilation or vasopressors) from March to August despite an overall increase in the average age of hospitalized patients [9]. This phenomenon may be due to experience gained from treating patients with COVID-19 and/or effective action taken by the VHA to meet care and resource demands, as evidenced by the positive transition in treatment modalities [24]. During our study period, we observed treatment with experimental drug regimens such as hydroxychloroquine decline to less than 1% by August, while use of more evidence-backed treatments such as dexamethasone and remdesivir significantly increased [8, 25–27]. These findings highlight the need for more cautious approaches to both observational research and treatment of a new disease, as it appears patients may have received potentially ineffective or harmful treatment based on conjecture and pre-print manuscripts.

In addition to older age, prior work has documented evidence of increased risk for SARS-CoV-2 infection and disease severity and mortality among patients with certain symptoms, pre-existing medical conditions, and abnormal laboratory findings [3, 9, 10, 20]. Although the significance and magnitude of associations varied between studies, our finding of declining mortality seems to support the idea of greater risk for severe or fatal disease among patients that have these risk factors. In our study, patients hospitalized in March compared to later months had more exacerbating symptoms (e.g., fever, dyspnea, cough) and evidence of biomarkers on inflammation, infection, cardiac and muscle injury, decreased liver and kidney function [3, 9, 10, 20]. Additionally, we found that both hospitalized and deceased patients had more underlying comorbidities in March compared to later months including severe cardiovascular diseases, diabetes, renal disease, and obesity. However, deceased patients had a higher frequency of such comorbidities. While more comorbidities may contribute to disease severity or mortality, they may not contribute to greater risk for SARS-CoV-2 infection as we did not find proportional declines in cases or hospitalizations. The specific reasons for the apparent decline in case severity and mortality are unclear, but may be due to increased dissemination of risk information and potentially greater compliance with public health measures among those with poorer overall health.

Lastly, studies have reported elevated hospitalization and mortality rates among black compared to white patients with COVID-19, citing differences in access and quality of care, more underlying comorbidities, and lower SES as potential reasons for these disparities [5, 7]. In our study, we observed a higher proportion of hospitalizations and in-hospital deaths among black compared to white patients in March, similar proportions of black and white patients in April, and notably fewer black patients from May through August. However, black patients consistently had a hospitalization and 30-day in-hospital mortality rate that was more than two times their overall veteran population share of 12% throughout the study period [28]. The initially higher rate of hospitalization among black patients with COVID-19 in our study could potentially be explained by structural confounding in which more black patients reside in densely populated urban areas that were also more likely to be affected during the first outbreak of SARS-CoV-2 [4, 9]. Additionally, more black patients may work in service and essential industries that do not allow work from home and create challenges for social distancing [4]. It is possible that the declining rate of 30-day in-hospital mortality among black patients with COVID-19 in our study could be due to the fact that unlike the general U.S. population, all patients treated within the VHA have insurance coverage and could readily seek high quality health care and treatment services if needed [4, 9, 29]. These results appear to align with another national cohort study of 16,317 VHA patients diagnosed with COVID-19 between February 2020 and July 2020 [4]. The authors found evidence of greater risk for contracting SARS-CoV-2 among black compared to white patients, but no difference in 30-day mortality after adjusting for patients’ sociodemographic and clinical characteristics [4]. Therefore, in addition to bolstering the capacity of health care systems and implementation of strict public health measures, ensuring equitable care access should be a central goal of COVID-19 mitigation efforts [4, 6, 9].

### Limitations

We acknowledge important limitations to our study. The VHA typically treats a population that is older, medically complex, and have greater risk behaviors compared to the general U.S. population [30]. However, prior work has found no evidence of differences in comorbidity burden between VHA and non-VHA users after controlling for age, sex, race, geographic region, and urbanicity of residence [17]. Additionally, the frequency and type of comorbidities identified in this study are similar to those reported among non-VHA patients hospitalized with COVID-19 [3, 11]. Therefore, our findings may still be generalizable to the larger U.S. population. We reported the month-to-month and cumulative disease burden on the VHA, which may not account for regional differences in sociodemographic characteristics, government restrictions and policies, or the magnitude and timing of case peaks. Future work on the regional intensity and timing of outbreaks may be beneficial for identifying specific areas with elevated disease burden and help inform allocation of limited resources.

## Conclusions

Our study provides greater insight on the longitudinal trends in COVID-19 case rates and clinical management within a national health care system. Among patients that were hospitalized with COVID-19 between March and August 2020, we observed a trend towards decreased disease severity and mortality over time, despite increases in cases, hospitalizations, and average age. This may be explained by the implementation of public health measures, as well as improved management of COVID-19 due to the introduction and approval of dexamethasone and remdesivir.

## Supporting information

S1 Checklist STROBE statement—checklist of items that should be included in reports of cohort studies. (DOCX)
